# FBH1 and the replication stress response: Implications for genome stability and cancer development

**DOI:** 10.1016/j.dnarep.2025.103865

**Published:** 2025-06-26

**Authors:** Joshua L. Turner, Jennifer M. Mason

**Affiliations:** Department of Genetics and Biochemistry, Clemson University, United States

**Keywords:** FBH1, replication stress, cancer

## Abstract

The replication stress response plays important roles in maintaining genome stability.In this review article, we focus on the role of FBH1 in the replication stress response and promoting death in cells with excessive DNA damage. FBH1-deficiency results in resistance to replication stress. We discuss how loss and gain of FBH1 in a wide variety of cancers can contribute to tumor-associated phenotypes, impact cancer therapies and be exploited for potential targeted therapies.

## 1. Introduction

Replication forks encounter many obstacles such as DNA secondary structures, DNA lesions, or protein complexes that can slow and/or stall replication fork progression. Any impediment that results in slowing or stalling of the replication fork machinery is collectively known as replication stress. To combat replication stress, cells have elaborate responses to halt cell cycle progression, repair underlying damage, and bypass lesions to allow replication to continue. Cancer cells are prone to high levels of replication stress, making them dependent on the replication stress response. As a result, targeting the replication stress response is an attractive strategy for cancer therapies.

FBH1 has many roles in DNA repair. FBH1 is a member of the UvrD helicase family with 3’ to 5’ helicase activity primarily functioning on the lagging strand during DNA synthesis [1,2]. Unique among helicases, FBH1 is the only known helicase with an F-Box domain and interacts with SKP1 and Cullin to form the SKP1/Cullin/F-box (SCF) complex [2, 3]. The SCF^Fbh1^ complex is an E3 ubiquitin ligase and the only known target for FBH1-mediated ubiquitination is RAD51 [3,4]. FBH1 homologs containing both DNA unwinding and E3 ubiquitin ligase activity are only found in *Schizosaccharomyces pombe* and vertebrate species [5,6]. In DNA repair, FBH1 has been shown to promote fork regression, double strand break (DSB) formation at stalled forks, negatively regulate recombination, and promote resolution of decatenation stress. In this review, we discuss how FBH1 functions to protect cells from genome instability and how mis-regulation of FBH1 can contribute to cancer.

## 2. FBH1 plays critical roles at stalled replication forks

### 2.1. FBH1 promotes DSB formation at stalled replication forks

Prolonged replication stress results in excessive ssDNA accumulation depleting the cellular pool of RPA. Exhaustion of the available RPA pool results in wide-spread DNA breakage (aka replication fork collapse) characterized by high levels of RPA S4/8 and γH2AX [7]. After prolonged stress, FBH1 promotes DSB accumulation and replication stress sensitivity [8,9]. FBH1 works in concert with the MUS81 endonuclease to cleave stalled replication forks resulting in DSB formation [9]. FBH1-deficient cells display a significant reduction in phosphorylation of signaling pathways controlled by ATM and DNA-PK, as well as their downstream targets, most notably pRPA S4/8, p53 and γH2AX likely due to the loss of DSB formation [8,10]. FBH1-deficient cells exhibit increased resistant to replication stress induced by hydroxyurea (HU), ultra-violet irradiation (UV), and aphidicolin [3,8,9,11]. It is still unclear how FBH1 promotes the replication stress response and sensitivity to replication stress, but it appears the role of FBH1 may be species specific. Human U2OS cells deficient in FBH1 have increased resistance to UV induced damage, while similar studies in *FBH1*^*−/−*^ chicken and fbh1Δ yeast cells show either no change or an increase in UV sensitivity, respectively [6,12,13]. In yeast and chicken cells, Fbh1 resolves recombination intermediates, but a role in promoting DSBs at stalled replication forks has not been reported [12,14]. One possibility is FBH1 has evolved replication-specific roles in mammalian cells that are required to promote replication stress sensitivity. Survival studies across species using a wider range of genotoxic agents are required to determine if FBH1 has species-specific roles in the replication stress response.

### 2.2. FBH1 is recruited to DNA damage by PCNA

Localization of FBH1 to sites of UV-induced DNA damage is facilitated by the Proliferating Cell Nuclear Antigen (PCNA) [15]. After targeted UV radiation, mono-ubiquitinated PCNA was shown to co-localize with FBH1 at the sites of damage. FBH1 contains two independent PCNA interacting domains critical for localization. FBH1 contains a PCNA-interacting protein-box (PIP box) located in the disordered N-terminus region and an APIM motif located within the UvrD helicase domain of the C-terminus [15]. Cells expressing either FBH1 APIM mutants or PIP mutants exhibit reduced localization to sites of UV-induced damage, but cells expressing FBH1 containing mutations in both domains results in complete loss of FBH1 localization [15]. These data suggest that both the APIM and PIP domains are required for the PCNA interaction and are essential for the recruitment of FBH1 to DNA. Structurally, the PIP and APIM domains on FBH1 were found to bind to the same hydrophobic pocket on PCNA [16]. Alpha fold modeling suggests that FBH1 can bind to a PCNA trimer at both PIP and APIM domains simultaneously. Cells expressing a PIP-domain FBH1 mutant did not exhibit a decrease in DSB signaling [8]. One possibility is that the interaction with PCNA via the APIM motif was sufficient for FBH1 to carry out functions. Consistent with this, mutations in both APIM and PIP motifs was required to disrupt the anti-recombinase activity of FBH1 [15].

Polyubiquitination of FBH1 by the ${\text{PCNA-CRL}}_{4}^{\text{cdt2}}$ pathway initiates degradation of FBH1 [15]. Similar to other PCNA interacting proteins ${\text{CRL}}_{4}^{\text{cdt}2}$ interacts and promotes degradation of FBH1 through its PIP degron motif after UV-radiation. Mutations to the FBH1 PIP degron increases FBH1 stability following UV radiation, as well as inhibits recruitment of polη to sites of DNA damage. It is unclear whether FBH1 itself inhibits polη recruitment or if it is due to competition in PCNA binding [15].

### 2.3. FBH1 mediates fork regression at stalled replication forks

**Fig. 1.**
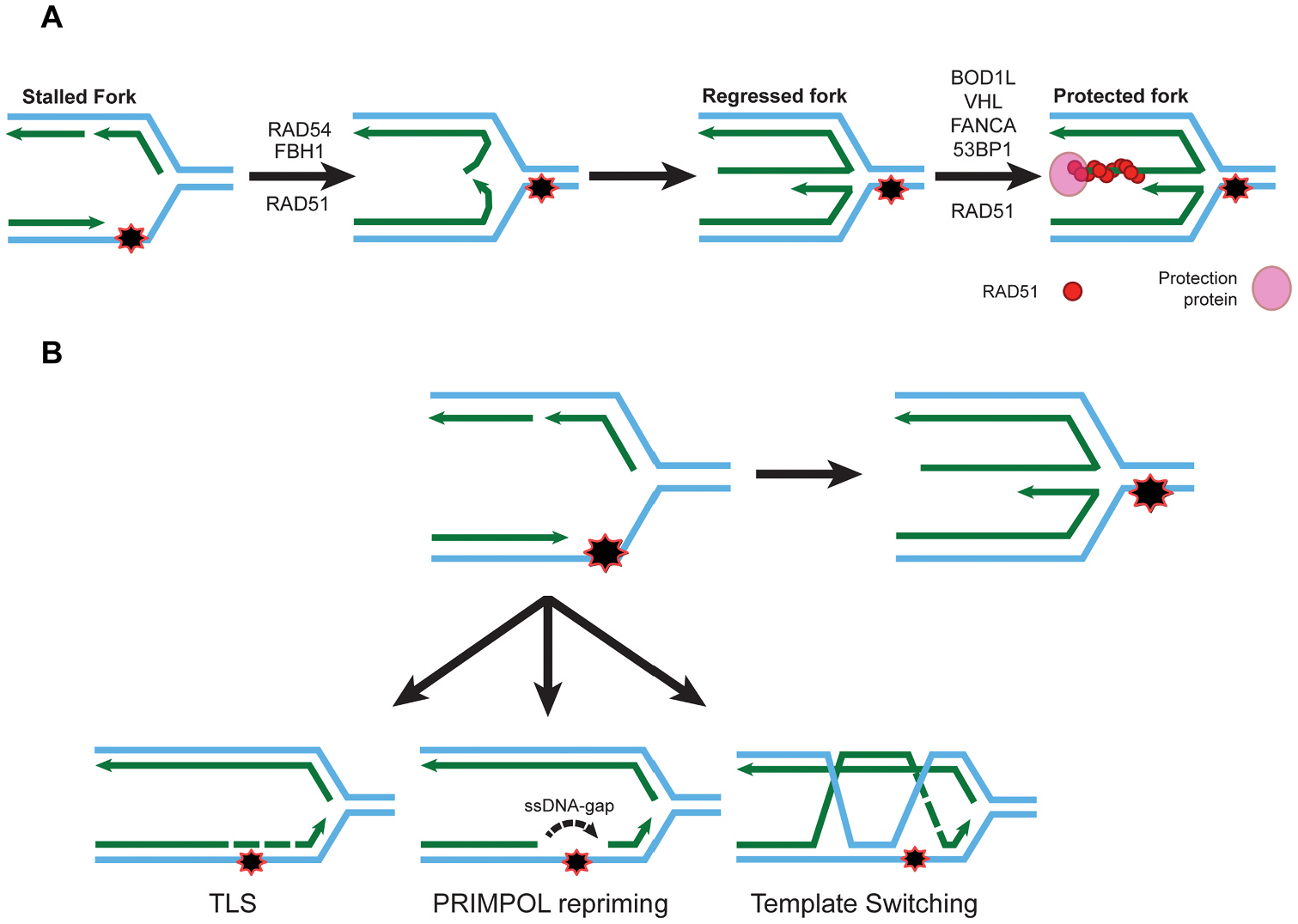
Activity of FBH1 in DNA repair. (A) Diagram of FBH1 mediated fork regression. At stalled replication forks FBH1, RAD51, and RAD54 promote fork regression by reannealing the nascent DNA strands together forming a Holliday junction like intermediate. RAD51 is loaded into the newly annealed regressed fork and stabilized by enzymes known as protection enzymes. FBH1 mediated regressed forks are known to be protected by BOD1L, VHL, FANCA, and 53BP1. (B) Unrestrained fork progression in the absence of fork regression has been attributed to alternative DNA damage tolerance pathways. TLS polymerases can replicate through DNA lesions inserting predetermined nucleotides opposite unreadable nucleotides. PRIMPOL can reprime downstream of DNA lesions producing a series of ssDNA gaps that require post replication repair. Finally, a lesion can be bypassed by RAD51 dependent template switching using the genetic information from the sister chromatid to replicate past a DNA lesion.

In response to stress, replication forks are remodeled in a process known as fork regression (aka fork reversal). During this process, nascent strands are unwound and annealed to convert a replication fork from a 3-way to a 4-way junction that resembles a Holliday junction (Fig. 1A). Regressed forks are critical structures that protect genome stability. Fork regression protects DNA from nucleolytic degradation that can occur at stalled replication forks. The central mitotic homologous recombination enzyme, RAD51 binds to the regressed arm to protect from degradation by nucleases such as MRE11, EXO1, and DNA2 [17,18]. FBH1 has been shown to mediate fork regression in response to HU and this activity requires the helicase activity, but not the ubiquitin ligase domain [19,20].

FBH1-mediated fork regression is genetically distinct from fork regression mediated by HLTF, SMARCAL1, and ZRANB3 and the role of FBH1 in promoting fork reversal has been confirmed by electron microscopy [19]. FBH1-mediated fork regression requires the branch migration activity of RAD54L [21]. Forks regressed by FBH1-dependent pathway require a unique set of fork protection proteins including BOD1L, 53BP1, FANCA, and VHL [20,22]. Together, these results indicate that the HLTF, SMARCAL1, ZRANB3 or FBH1 mediate distinct replication fork reversal pathways. However, the underlying differences between the two pathways is still not clear.

Cells harboring defects in fork reversal, including HLTF and RAD54, exhibit unrestrained replication fork progression [21,23,24]. In cells with defects in HLTF-dependent fork reversal, replication progresses even in the presence of DNA damage. Unrestrained replication in HLTF-deficient cells has been attributed to two other DNA Damage Tolerance (DDT) pathways (Fig. 1B). Translesion synthesis (TLS) uses specialized DNA polymerases that bypass lesions preventing prolonged replication fork stalling and collapse [25,26]. TLS polymerases lack proof reading domains and are more error prone [27]. Primase-polymerase (PRIMPOL) bypasses DNA lesions by repriming downstream of a stalled replication fork and inserts a couple of nucleotides to allow replication to resume by the replicative polymerases [28]. Increased PRIMPOL activity results in the accumulation of ssDNA gaps that must be filled with TLS polymerases or template switching prior to mitosis [29]. PRIMPOL, like TLS, is an error prone DDT pathway lacking proofreading activity [30]. In HLTF-deficient cells, replication fork progression is PRIMPOL-dependent. However, cells containing mutations in the HIRAN domain of HLTF promote cell progression in a TLS dependent manner indicating different activities of HLTF inhibit different DDT pathways [23].

Unrestrained fork progression occurs in the absence of FBH1 in cells treated with a low dose of HU [21]. However, the pathway that is responsible for continuing replication and whether different functions of FBH1 have different effects on DDT pathway usage has not been elucidated. We propose that FBH1, like HLTF, is preventing replication by more than one mechanism. In response to HU, FBH1-deficient cells exhibit a decrease in RPA loading on chromatin that is detectable by western blotting, but not by immunofluorescence indicating that the same percentage of cells accumulate ssDNA in control and FBH1-deficient cells, but there is less ssDNA accumulation and RPA loading per cell in the absence of FBH1 [8]. Interestingly, a cell line expressing the helicase dead mutant of FBH1 exhibited a substantial defect in RPA loading at HU-stalled forks compared to FBH1 deficient cells [3]. This result suggests that the presence of non-functional FBH1 (i.e., helicase dead) is inhibitory to RPA loading at HU-stalled replication forks. The authors proposed that FBH1 may have a pro-recombination role by promoting ssDNA formation at stalled replication forks. However, given the recent studies that have shown an increase in TLS or PRIMPOL results in a reduction in RPA accumulation, we propose the defect in RPA loading is due to unrestrained replication resulting in less RPA accumulation at stalled forks [23].

The loss of FBH1-mediated fork reversal can result in reduced DSB accumulation and increased survival in response to HU and UV by multiple mechanisms. First, increased activity of one or more DDT pathways can contribute to the increased survival of FBH1-deficient cells following UV and HU treatment. Increased DDT activity has been linked to chemotherapeutic resistance. Increased PRIMPOL activity has been shown to result in cisplatin-resistance in BRCA1 deficient cells [31]. Furthermore, PRIMPOL partially contributes to increased survival of HLTF-deficient cells by promoting S phase progression in the presence of HU. However, HTLF loss reduced DSB accumulation and DSB signaling in PRIMPOL knockout cells suggesting that HLTF promotes DSB accumulation by a mechanism independent of PRIMPOL. In addition, cells expressing HLTF containing a mutant in the HIRAN domain, known to be important for fork regression, inhibited replication fork progression by REV1-mediated TLS suggesting different activities of HLTF can limit different DDT pathways in response to HU [23]. Second, FBH1 may generate an intermediate susceptible to cleavage by endonucleases such as MUS81. Consistent with this, regressed forks that undergo nascent strand degradation in BRCA2-deficient cells, due to oncogene-induced replication stress can be susceptible to cleavage by MUS81 [17,32]. However, it is unlikely that fork regression itself is required for DSB break accumulation and decreased survival as depletion of two fork remodelers, ZRANB3 and SMARCAL1, results in increased sensitivity to HU [33,34]. An alternative possibility is the role of FBH1 in promoting DSB formation at stalled forks is independent of a role in fork regression. For instance, FBH1 could be required for proper localization of MUS81 to stalled replication forks. A future area of study will be to determine how different DDT pathways are contributing to increased survival in FBH1-deficient cells and cells lacking various activities of FBH1 to determine how FBH1 promotes death in cells with excessive DNA damage.

### 2.4. FBH1-deficient cells recover quickly from replication stress

After release from HU, FBH1-deficient cells recover faster from replication stress [11,19]. One likely reason is that the reduction in DSBs in FBH1-deficient cells allow cells to resolve underlying replication intermediates quickly. After release from HU treatment, FBH1-deficient cells progress to mitosis faster than control cells and accumulate 53BP1 nuclear bodies in G1 indicating FBH1-deficient cells enter mitosis with under-replicated DNA [19]. Another possibility is the shorter ssDNA tracts, or gaps accumulating in FBH1-deficient cells due to increased PRIMPOL activity at stalled forks are escaping detection prior to mitosis leading to increased under-replicated DNA in G1 [35]. Future work will need to be done to test this model and determine if faster recovery is solely due to a reduction of DSBs, or if the faster recovery from HU-induced replication stress in the absence of FBH1 is independent of DSB formation.

### 2.5. FBH1 negatively regulates homologous recombination

Homologous recombination (HR) is highly regulated to prevent unwanted and unscheduled recombination. One regulatory mechanism is to dismantle RAD51 pre-synaptic filaments to prevent homology search and strand exchange. One of the most characterized anti-recombinases that functions to dismantle RAD51 filaments is the yeast UvrD helicase, Srs2 [36].

In *S. pombe*, Fbh1 (spFbh1) limits accumulation of recombination intermediates using both the helicase and ubiquitin ligase activities [10, 13,14]. Similar to Srs2 mutants, SpFbh1-deficient strains have an increase in Rhp51 (Rad51 in *S. pombe*) focus formation [6,13,14]. Fbh1 prevents accumulation of toxic recombination intermediates in cells lacking other anti-recombinases including Rqh1 and Srs2 and viability is restored by deletion of Rhp51 [6,13]. Overexpression of Fbh1 limited recombination resulting in reduced Rad51 focus formation and hyper-sensitivity to genotoxic stress [14]. Together, these results indicate that Fbh1, like Srs2, functions to restrain HR in cells.

The extent that FBH1 regulates HR in vertebrate cells is not as clear. An increase in spontaneous RAD51 foci in FBH1-deficient cells has been observed in mouse and human cells consistent with a role of FBH1 in regulating RAD51 and HR, but normal Rad51 kinetics were observed in *FBH1*^*−/−*^ DT40 cells [3,12,37,38]. However, there have been conflicting results regarding the impact of FBH1 status on recombination levels. Some studies have reported an increase in recombination levels in FBH1-deficient cells using the DR-GFP reporter assay, others have only observed an increase in recombination levels using the sister chromatid exchange (SCE) assay and some have observed no difference in the DR-GFP assay [3,12,37,38]. The DR-GFP assay measures synthesis-dependent strand annealing (SDSA) whereas SCE requires a crossover event. The increase in recombination observed in Fbh1-deficient mouse ES cells in one study only using the DR-GFP assay led to the authors to propose that Fbh1 is only regulating SDSA [37]. However, others have observed a significant increase in spontaneous SCEs in FBH1-deficient cells in chicken and human cells suggesting a larger role in regulating HR [3,12]. The underlying biological difference resulting in contradictory results is not clear. One possibility is FBH1 limits recombination specifically at stalled or collapsed recombination forks, so hyper-recombination is only observed under specific conditions. Consistent with this, yeast Fbh1 was shown to limit recombination at blocked and collapsed replication forks [14]. It will be of interest to measure recombination using SCEs after different replication stress inducing drugs such as HU treatment and UV irradiation. It is important to note that purified FBH1 removes RAD51 from DNA, overexpression of FBH1 reduces RAD51 focus formation and inhibits HR, and FBH1-deficient cells have increased RAD51 focus formation which are consist with a role of FBH1 in playing a significant role in regulating RAD51 and HR [3,12–14,37,38].

**Fig. 2.**
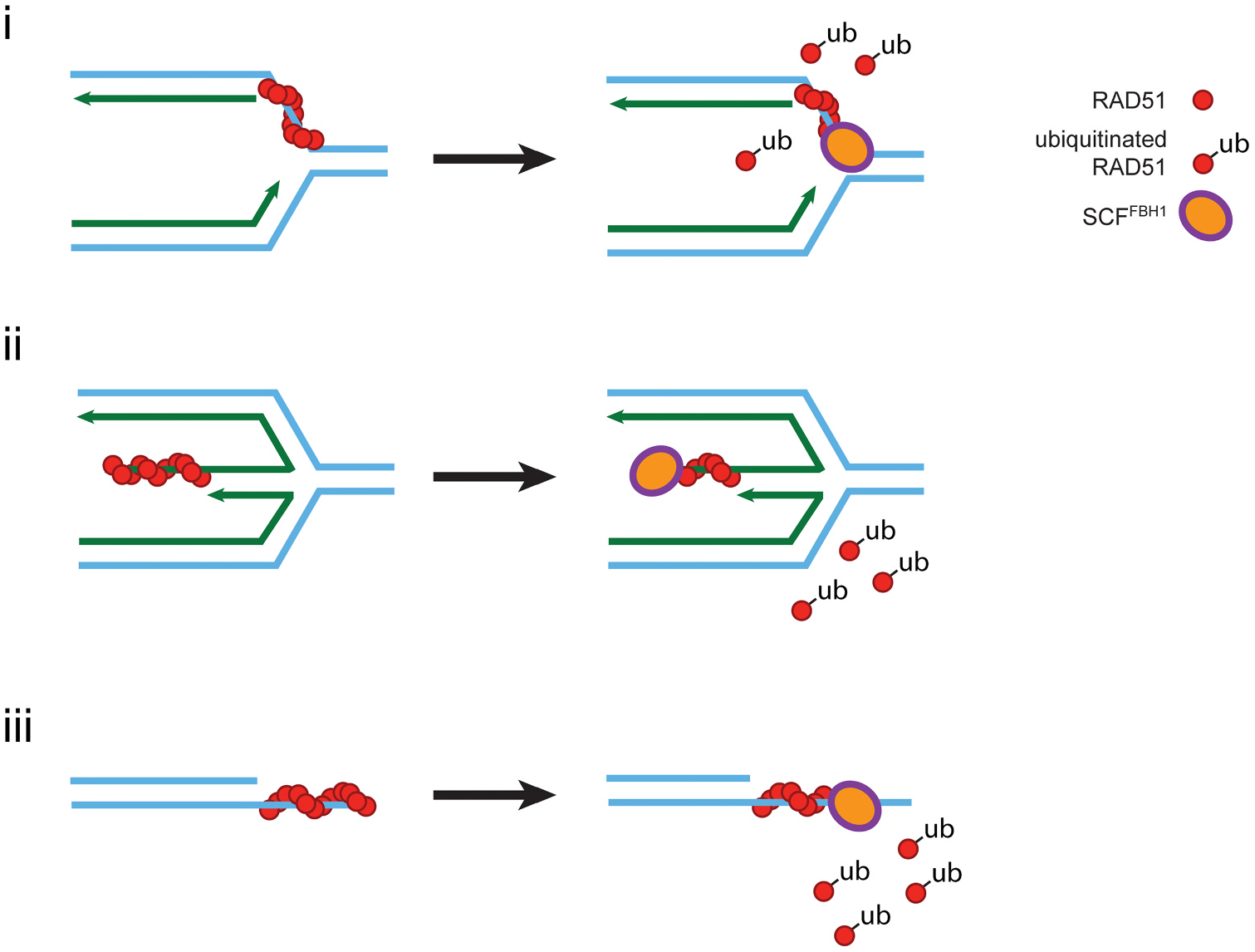
FBH1 removes and ubiquitinates RAD51 on ssDNA. Based on the known biochemical activities of FBH1, removal can occur at 3 different intermediates. (i) FBH1 apart of the SCF complex can remove RAD51 from ssDNA at replication forks. (ii) SCF-FBH1 can remove RAD51 from the single-stranded end of a regressed replication fork. (iii) SCF-FBH1 can remove RAD51 from the terminal end of a regressed DSB end.

The anti-recombinase function of FBH1 requires both the helicase and the ubiquitin ligase activity [19]. FBH1 directly interacts with RAD51 and removes RAD51 from ssDNA [1,3,37]. Once RAD51 is removed from ssDNA, the SCF^FBH1^ complex ubiquitinates RAD51 to prevent re-association to ssDNA [39] (Fig. 2). It is not clear if removal of RAD51 occurs before ubiquitination or if the two steps occur simultaneously in the cell. In biochemical experiments, the formation of SCF^FBH1^ does not interfere with either the DNA binding activity or the helicase activity of FBH1 suggesting that removal and ubiquitination may be linked in the cell [40].

Does the anti-recombinase activity of FBH1 promote cell death in response to replication stress induced by HU? In cells, expression of a ubiquitin resistant RAD51 mutant resulted in a reduction of DSB formation and increased resistance to HU suggesting unregulated RAD51 is resulting in replication stress resistance in the absence of FBH1 [37]. However, the impact of these mutations on the biochemical activities of RAD51 was not tested, so it cannot be ruled out that mutations of these residues had an impact on RAD51 function in cells independent of FBH1. Cells expressing FBH1 with a mutated F-box, that disrupts the interaction with the SCF ubiquitin ligase complex, are not resistant to replication stress and are indistinguishable from control cells in the replication stress response [8]. Although these results seem contradictory, the ubiquitin ligase activity of FBH1 is also used for self-regulation [1]. In biophysical experiments, ubiquitinated FBH1 is unable to interact with RAD51 preventing removal from ssDNA. In an F-box mutant, FBH1 would still retain the ability to remove RAD51, but would not be able to prevent re-binding of RAD51 on DNA by ubiquitination. However, FBH1 would not be able to self-ubiquitinate to turn off the anti-recombinase activity potentially resulting in a constitutively active anti-recombinase. Consistent with this, over-expression of FBH1-F-box mutant resulted in a decrease in RAD51 focus formation suggesting the ubiquitin ligase activity is not required for anti-recombinase activity [3].

To determine how FBH1 regulates HR in human cells, it is imperative to identify a mutant that only disrupts the anti-recombinase activity of FBH1. One potential strategy would be to disrupt the interaction between FBH1 and RAD51. The anti-recombinase activity of FBH1 depends on a direct interaction between FBH1 and RAD51 [1]. Mutations that disrupt the interaction between RAD51 and other anti-recombinases including srs2 and RECQ5 have provided powerful tools to study the biological function of RAD51 removal in cells [41–44]. For instance, an anti-recombinase mutant of FBH1 can be used to determine if FBH1 removes RAD51 from 3-way replication forks, regressed forks, and/or DSBs (Fig. 2). However, it remains possible that loss of this interaction may also disrupt the fork regression activity of FBH1 as RAD51 functions in the FBH1-dependent fork regression pathway [20]. Determining the biological importance of the FBH1-RAD51 interaction in cells can provide insights into the role of FBH1 at stalled replication forks.

## 3. FBH1 may have additional functions in DNA repair

FBH1 may also have additional roles in maintaining genome stability during mitosis. FBH1-deficient mouse cells and cells containing a deletion of the FBH1-F-box domain exhibit reduced survival in response to decatenation stress induced by the topoisomerase II inhibitor, ICRF-193 [38]. Although the mechanism has not been elucidated, FBH1 appears to play a role in maintaining proper anaphase separation following induced decatenation stress suggesting FBH1 may be required to resolve repair intermediates prior to mitosis [38]. The F-box mutant displayed similar phenotypes suggesting that SCF^FBH1^ ubiquitination of target proteins may be required for resolution [38]. An interesting future area of research will be to determine if FBH1 plays a direct role in resolving mitotic stress or if the increase in sensitivity is due to the roles of FBH1 at stalled replication forks. One possibility is unresolved recombination or replication intermediates are persisting in FBH1-deficient cells resulting in mitotic defects.

## 4. FBH1 is mis-regulated in cancer

### 4.1. FBH1 loss in melanoma

FBH1 plays a critical role in promoting death in cells with excessive damage after replication stress induced by HU treatment and UV irradiation [8,9]. One study aimed to determine how loss of FBH1 contributed to melanoma [11]. Hemizygous or homozygous deletions that included *FBH1* were found in up to 36 % of primary melanoma and 63 % of metastatic melanoma [11]. Interestingly, one melanoma cell line harbored the same mutation, D698N, used in cell experiments to disrupt the helicase activity.

There are several lines of evidence that reduced FBH1 expression in melanoma disrupted the replication stress response and may contribute to melanoma formation [11]. Melanoma cell lines with reduced FBH1 protein levels exhibit lower levels of DSB signaling after HU treatment as indicated by pRPA2 S4/8, γH2AX, and p-P53 Ser15. These results indicate that the reduced levels of FBH1 in melanoma cells is sufficient to impede activation of ATM and DNA-PKcs dependent signaling. Increasing the levels of FBH1 in cells rescued the defect in ATM and DNA-PKcs dependent signaling. Finally, melanocytes depleted of FBH1 expression exhibited increased growth using the anchorage-independent assay after UV irradiation suggesting FBH1 protects cell from UV-mediated malignant transformation.

### 4.2. Copy number changes in FBH1 are common in a variety of cancer types

**Fig. 3.**
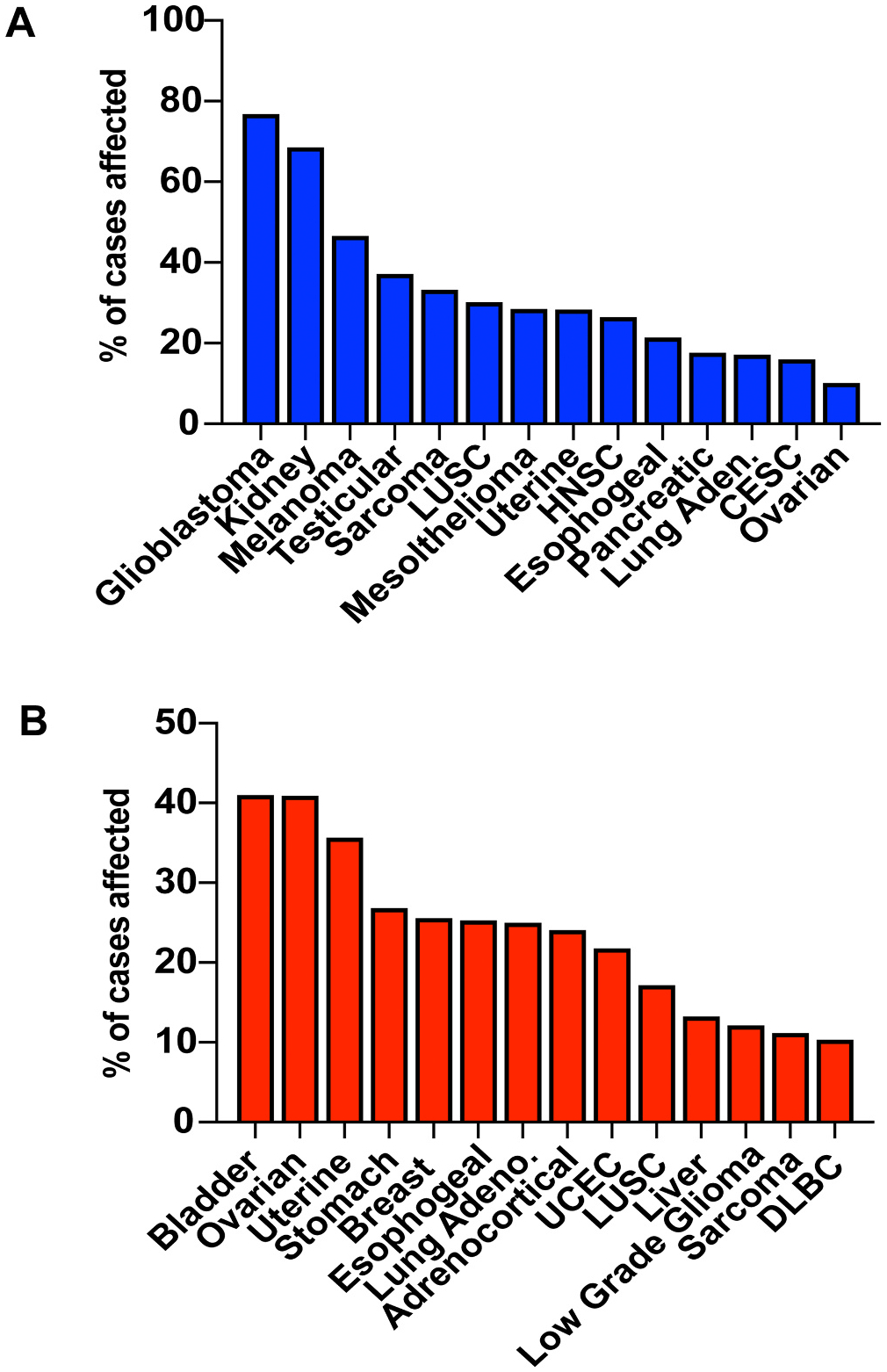
FBH1 is mis regulated in a high percentage of cancers. Bar graphs depict percentage of cancer types with (A) FBH1 copy number loss (B) FBH1 copy number gain. Abbreviations are LUSC, Lung squamous cell carcinoma; CESC, Cervical squamous cell carcinoma and endocervical adenocarcinoma; HNSC, Head and Neck squamous cell carcinoma; UCEC, Uterine Corpus Endometrial Carcinoma; DLBC, Lymphoid Neoplasm Diffuse Large B-cell Lymphoma. The results shown here are in whole based upon data generated by the TCGA Research Network: https://www.cancer.gov/tcga.

The impact of FBH1 status in other cancers is understudied. *FBH1* mutations have been identified in a wide variety of cancers, but FBH1 is not characterized as a cancer driving gene [45]. The most common mutations identified in FBH1 are missense mutations, but recurring mutations in specific tumor types were not observed [45]. Therefore, functional validation will need to be conducted to determine how these variants impact FBH1 function. However, TCGA analysis demonstrates that copy number loss of *FBH1* is common in a wide variety of cancer types and ranges from 10 percent in ovarian cancer to 77 percent in glioblastoma (Fig. 3A). It is not clear how the copy number changes translate to change in FBH1 protein levels, but the results from melanoma cell lines suggest that even a modest reduction in FBH1 is sufficient to disrupt the replication stress response and lead to increased cellular survival after HU treatment [11]. To better understand how FBH1 loss plays a role in cancers, additional studies need to be conducted to determine how reducing levels of FBH1 protein impacts the various functions of FBH1 and to determine how copy number losses observed in different cancer types disrupt FBH1 function.

Given the roles of FBH1 at replication forks, there are multiple ways FBH1 loss can contribute to cancer. First, FBH1-mediated fork regression may restrain replication progression by DDT pathways including PRIMPOL and TLS. DNA synthesis mediated by PRIMPOL and TLS is more error prone, so increased activity would lead to increased mutagenesis in FBH1-deficient cells [46,47]. Second, FBH1 kills cells with excessive DNA damage in response to a variety of replication stress-inducing drugs including HU and UV irradiation [3,8,9,11]. A failure to kill cells with excessive DNA damage can result in increased mutagenesis that can contribute to tumor development. As observed in melanocytes, the role of FBH1 in promoting DSB formation and apoptosis protects cells from UV-mediated malignant transformation [11]. Finally, cancer is associated with high levels of replication stress due to uncontrolled cell division and oncogene-induced replication stress [48]. In this context, FBH1 loss may result in a higher tolerance to replication stress in cancer cells facilitating increased proliferation and survival.

Copy number gains were also identified in a wide variety of cancers and ranged from 10 percent in B-cell lymphoma to 40 percent in bladder and ovarian cancer (Fig. 3B). The impact of *FBH1* copy gain in these tumor types has not been investigated. Overexpression of FBH1 results in premature removal of RAD51 from DNA damage sites after irradiation resulting in HR-deficiency [3]. It will be of interest to determine if copy number gain of FBH1 in tumor types impacts RAD51 localization and HR efficiency. In this case, tumors with FBH1 copy number gain may be hyper-sensitive to specific treatments including olaparib and cisplatin [49,50].

### 4.3. FBH1 deficiency as a target for chemotherapeutics

There are at least two potential ways FBH1 status may impact chemotherapeutic response. First, FBH1 status may impact the response to chemotherapeutics that kill cells by inducing lethal levels of replication stress. Certain inhibitors such as those that target ATR and CHK1 result in high levels of replication stress overwhelming the cellular machinery leading to widespread DSB formation [7,51]. Given the role of FBH1 in promoting DSBs in response to UV irradiation and hydroxyurea, a reduction in FBH1 may also be required for DSB formation after treatment with replication-stress inducing inhibitors. Second, the replication stress response defects in FBH1-deficient cells may be a potential chemotherapeutic target. Recently, we demonstrated FBH1-deficient cells exhibit increased sensitivity to WEE1 kinase inhibitors [52]. WEE1 inhibition leads to replication catastrophe but also disrupts the G2/M checkpoint killing cells by forcing cells into mitosis with unresolved DNA damage. As expected, FBH1-deficient cells exhibited a dampened replication stress response in response to WEE1 inhibition consistent with a role of FBH1 in promoting replication fork cleavage. However, FBH1-deficient cells exhibited a significant increase in multi-nucleation indicative of mitotic catastrophe [52]. Together these data suggest that although FBH1-deficient cells did not respond properly to WEE1 inhibition as indicated by the reduced replication stress response, FBH1-deficient cells are more dependent on the G2/M checkpoint. We propose that FBH1-deficent cells rely on post-replication repair in G2 to resolve replication intermediates that arise due to loss of fork regression [52]. This study suggests that targeting the G2/M checkpoint combined with induced replication stress may be a viable strategy to target tumors with defects in FBH1. It is important to note that the initial study was performed in FBH1 knockout cells. Determining how cancer cell lines with reduced FBH1 respond to WEE1 inhibition is critical to determine how FBH1-deficient tumors respond to WEE1 inhibition.

The identification and characterization of targets that specifically target defects in FBH1-deficient cells has the potential to be effective in a wide variety of cancers (Fig. 3). A comprehensive understanding of how FBH1 loss impacts the replication stress response in cancer and how FBH1 functions in the replication stress response is important to identify potential chemotherapeutic strategies. We propose three main lines of questioning that will aid in the identification and characterization of strategies to kill FBH1-deficient tumor cells. First, identification of which DDT pathways are promoting replication fork progression in the absence of FBH1 is key to identifying potential strategies to target FBH1-deficiency in cancer. PRIMPOL-mediated repriming leaves ssDNA gaps behind the fork that are filled by post-replication repair pathways template switching and TLS [29]. Targeting replication gaps filling and repair is emerging as a potential chemotherapy [31]. Cancers that rely on gap synthesis by TLS are sensitive to REV1 inhibitors and it was demonstrated that the impact of TLS inhibitors is synergistic with ATR or WEE1 inhibitors [53]. Second, determining how FBH1 promotes cell death in response to different replication stress inducing drugs can identify strategies to restore DSB break formation killing FBH1-deficient cells. FBH1 promotes DSB accumulation and cell death in response to UV and HU treatment [8,9]. Fork regression occurs in response to a wide variety of genotoxic stresses, so it would be interesting to determine how FBH1 functions in response to other types of replication stress including chemotherapeutic drugs such as cisplatin and mitomycin C [53]. It is possible that some replication stress inducing drugs kill by a FBH1-independent mechanism. In this case, FBH1 status in cancer may be predictive of which replication stress inducing drugs will be effective in treating different cancer types.

Cancer cells already exhibit high levels of replication stress that leads to a dependence on the replication stress response to prevent replication catastrophe. Chemotherapeutic strategies targeting the replication stress response induce high levels of replication stress that is above the threshold that can be tolerated by cancer cells [54]. We propose FBH1 loss can be beneficial to cancer by raising the threshold of replication stress that can be tolerated by the cell. The identification of targets that restore replication-associated DSBs in FBH1-deficient cells could potentially identify targets that kill FBH1-deficient cells alone or in combination with replication stress inducing drugs. Finally, additional studies in relevant preclinical cancer cells lines need to be conducted to determine how FBH1 status in cancer impacts the replication stress response. To date, one study has been conducted to show FBH1 loss in melanoma is sufficient to result in increased survival in response to HU [11].

## 5. Concluding remarks

FBH1 plays a critical role in mediating the response to replication stress. FBH1 cooperates with MUS81 to kill cells with excessive amount of DNA damage. Recent studies have greatly increased our understanding of how FBH1 functions in the replication stress response, but many questions remain. What pathways promote replication fork progression in the absence of FBH1 and is increased activity of these pathways contributing to replication stress resistance? What is the function of RAD51 regulation in cells? How does copy number gain and loss impact FBH1 activity in cancer cells and does FBH1 status impact the replication stress response? Can underlying defects in cells due to FBH1 status be exploited as a chemotherapeutic strategy? Future work into the molecular mechanism involving FBH1 in response to different forms of replication stress will answer these questions and potentially uncover novel targets for treatment.

## Data Availability

No data was used for the research described in the article.

## Abbreviations:

ssDNA: single-stranded DNA
DSB: double strand break
DDT: DNA damage tolerance
HU: hydroxyurea
UV: ultraviolet radiation
PCNA: Proliferating Cell Nuclear Antigen
